# Complication of Invasive Molar Pregnancy with *Clostridium perfringens* Sepsis

**DOI:** 10.1155/2014/282141

**Published:** 2014-02-13

**Authors:** Sanmeet Singh, Kunal Angra, Bonnie Davis, Babak Shokrani

**Affiliations:** ^1^Howard University College of Medicine, Washington, DC 20059, USA; ^2^Department of Radiology, Howard University Hospital, Washington, DC 20060, USA; ^3^Department of Pathology, Howard University Hospital, Washington, DC 20060, USA

## Abstract

*Clostridium perfringens* (CP) is an anaerobic, Gram-positive bacillus associated with malignant diseases and near-term pregnancies. The necrotic tissue that results from these disease processes fuels the proliferation of CP, leading to gas gangrene and subsequently sepsis. Herein, we report a case of a 41-year-old female patient with a history of invasive molar pregnancy that was further complicated with a CP infection. Although past research has shown a link between *Clostridium* infection and choriocarcinoma (Chern-Horng and Hsieh, 1999), no previous cases of CP infection have been associated with invasive molar pregnancy. We also report complete resolution of the CP sepsis and its associated symptoms following the hysterectomy.

## 1. Introduction

*Clostridium perfringens* (CP), an anaerobic Gram-positive bacillus, is found among the normal human intestinal and vaginal flora in approximately 25% of healthy women [1]. *Clostridium* infections are commonly associated with malignant diseases [2, 3]. Along with malignancies, it is often associated with caesarean sections and incomplete pregnancies. Injured and necrotic tissue in the uterus after delivery permit bacterial incubation and overgrowth of bacterial colonies [4]. Occasionally, CP can progress to gas gangrene, a form of tissue death, and may eventually lead to sepsis [1].

## 2. Case Presentation

A 41-year-old woman with a two-month history of gestational trophoblastic disease was admitted to Howard University Hospital on October 28, 2012, complaining of severe abdominal pain with fever as well as vaginal bleeding with clots. Her vaginal bleeding occurred intermittently since her diagnosis of invasive molar pregnancy on August 1, 2012. Additionally, she experienced bilateral leg pain for which she was started prophylactically on 40 mg of enoxaparin (Lovenox) subcutaneously at the time of admission. However, sonography ruled out deep vein thrombosis of both of her legs. She had already received 2 cycles of IV EMACO chemotherapy (etoposide, methotrexate, actinomycin D, cyclophosphamide, and vincristine) by the time of presentation. At the time of her initiation of chemotherapy, her beta HCG (human chorionic gonadotropin) was 55,000 mlU/mL. Prior to this, she underwent a caesarean section with dilatation and curettage for her molar pregnancy at another institution on July 13, 2012. She was offered hysterectomy, which at the time she declined. At Howard University Hospital, she was diagnosed with anemia, recurrent carcinoma, sepsis, and hypertension. On physical examination, her temperature was normal, but she reportedly felt chills at home. She was started on metronidazole (Flagyl) 500 mg IV q 8 h., piperacillin/tazobactam (Zosyn) 3.375 g IV q. 6 h., and vancomycin.

**Figure 1:**
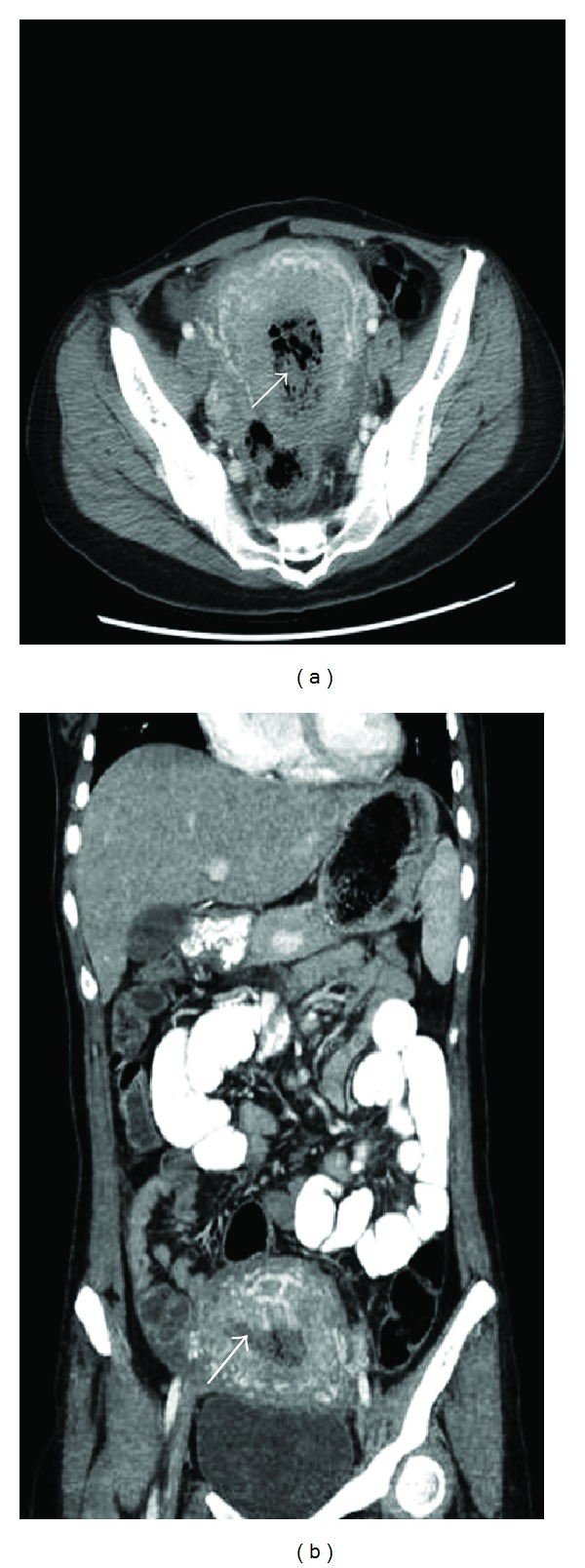
CT scan of abdomen and pelvis. Enlarged uterus with a markedly distended endometrial cavity contained a heterogeneous collection of soft tissue, debris, and gas bubbles (arrow).

She became febrile and developed hypotension on October 30, 2012. A computed tomography (CT) scan of the abdomen and pelvis showed an enlarged uterus measuring 16 cm × 10 cm with a markedly distended endometrial cavity which contained a heterogeneous collection of soft tissue, debris, and gas bubbles (Figure 1). There was hyperenhancement of the uterine wall vessels. There was no evidence of bowel obstruction or lymphadenopathy. The blood Gram stain showed large Gram-positive rods, confirmed to be CP by blood culture. She was diagnosed with *Clostridium perfringens* gas gangrene of the uterus and sepsis.

**Figure 2:**
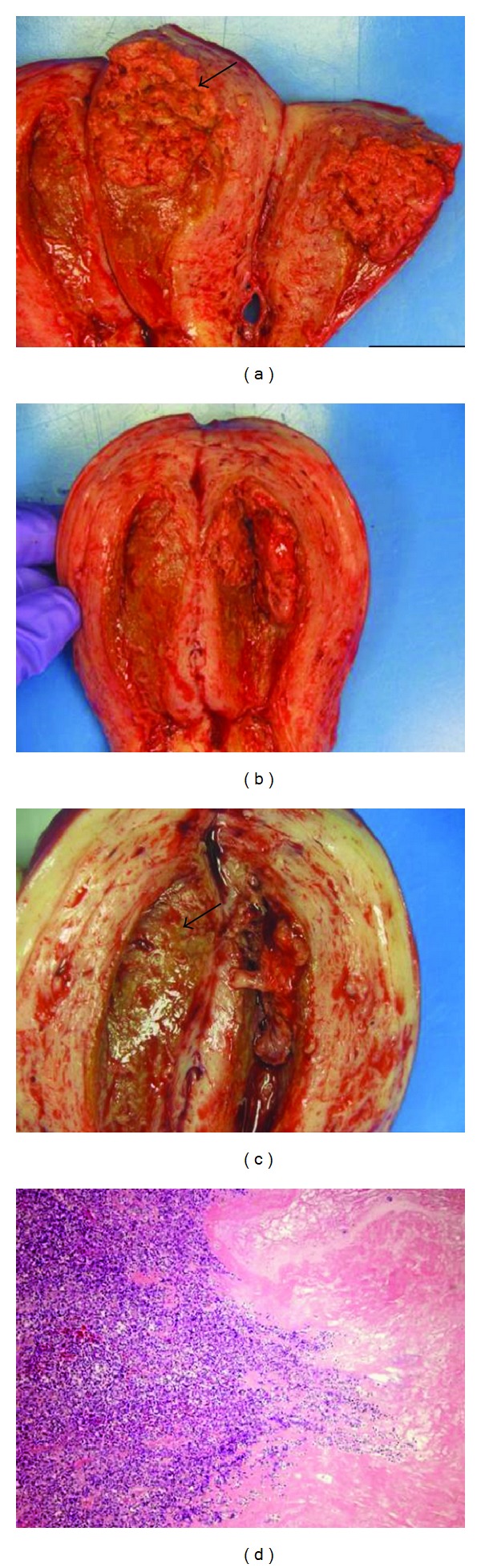
A tan fungating necrotic tumor in the anterior uterine wall (arrow) ((a) and (c)); low power microscopic section shows extensive necrosis and acute inflammation at the surface of tumor ((d) H&E, ×20).

**Figure 3:**
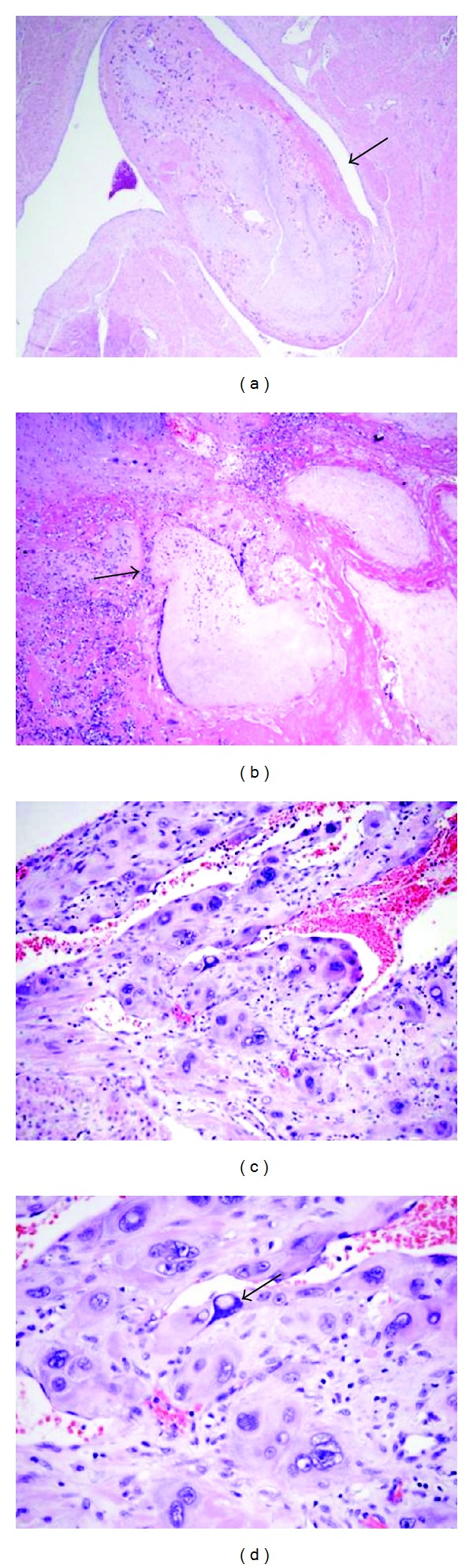
Hydropic villi invading into the myometrium and blood vessels (arrow) ((a) and (b) H&E, x40); markedly atypical trophoblastic proliferation with chemotherapy effect and exaggerated placental site (arrow) ((d) H&E, ×400).

She received an emergency total abdominal hysterectomy on October 31, 2012. Following the procedure, her beta HCG dropped to less than 10 mlU/mL. Gross examination of the uterus revealed a greenish/tan fungating necrotic tumor in the anterior uterine wall. On microscopic examination, it revealed an invasive hydatidiform mole with chemotherapy effect and exaggerated placental site. Also, acute endometritis was present in the background (Figures 2 and 3). She remained on antibiotics postoperatively for a few days. On a followup visit in February 2013, her beta HCG was less than 1 mlU/mL, and her edema had fully resolved.

## 3. Discussion

Gestational trophoblastic diseases (GTD) are a heterogeneous group of gestational and neoplastic conditions arising from the trophoblast. They include molar gestations and trophoblastic tumors. GTD varies widely among various populations with occurrences as high as 1/120 pregnancies in some areas of Asia and South America, compared to 0.6–1.1 per 1000 in the United States [5]. The incidence of hydatidiform moles is greater in women older than 40 years and younger than 20 years [6].

Hydatidiform moles arise from abnormal conceptions. Partial moles result from diandric triploidy, whereas complete moles result from diandry (fertilization of an empty ovum). Up to 50% of choriocarcinomas and 15% of placental site trophoblastic tumors follow complete moles. Most hydatidiform moles regress after suction evacuation, and the serum and urine HCG levels rapidly return to normal. Approximately 5–15% of patients with a hydatidiform mole progress to gestational trophoblastic neoplasia (GTN) [7]. GTN refers to evidence of trophoblastic activity after evacuation as shown by a stationary or rising HCG level.

Invasive moles are defined by the presence of hydropic villi from a hydatidiform mole invading into the myometrium, blood vessels, or extrauterine sites. They are found in about one-sixth of patients with complete moles. The patients usually have persistently high HCG levels and may present with uterine rupture. Chemotherapy and hysterectomy may be indicated. The diagnosis is usually made on a hysterectomy specimen.

The macroscopic appearance of an invasive mole varies with the extent of invasion. Variable numbers of molar vesicles may be seen in the endometrial cavity, myometrium, and adjacent extrauterine tissue. Significant hemorrhage is usually present. Microscopically, invasive moles exhibit molar villi within the myometrium or the blood vessels (Figure 3). Invasive moles need to be distinguished from choriocarcinomas; chorionic villi are absent in the latter. Invasive moles exhibit chorionic villi, even in metastatic foci.

Clostridia are facultative anaerobic Gram positive bacilli known to produce virulence through a dormant toxic state, called an endospore. *Clostridium perfringens* is a specific subtype that is commonly found in the vaginal flora of 1% to 27% of healthy women [1]. Occasionally, *Clostridium* infections can cause the potentially dangerous complication of gas gangrene which can ultimately lead to septic shock. Necrotic tissue associated with neoplasia may serve as a source of *Clostridium* infection and sepsis [8–10]. Ischemic and hypoxic conditions caused by rapid tumor cell growth create the anaerobic conditions necessary for *Clostridium* growth and spore germination [8, 11, 12]. Chern-Horng and Hsieh discovered an association between *Clostridium* infections and choriocarcinoma; this was the first reported involvement of gestational trophoblastic disease with *Clostridium* sepsis [2]. However, no previous cases of *Clostridium* infection have been associated with invasive molar pregnancy.

In our case study, we found a possible association between invasive molar pregnancy and CP sepsis. Complications of the invasive molar pregnancy with CP included gas gangrene and sepsis. We hypothesize that the CP bacteria originated from the vaginal flora and ascended into the cervix of the uterus, establishing the primary site of infection. The subsequent gas gangrene which led to sepsis resolved post-hysterectomy, suggesting that the invasive molar pregnancy located in the uterus was the source of the *Clostridium* infection. Likewise, the signs and symptoms of sepsis also resolved postoperatively.

## 4. Learning Points

- In the presence of invasive molar pregnancy, there is the possibility of the unusual and rare complication of *Clostridium* sepsis.
- In our case, the infection and sepsis subsided after hysterectomy, suggesting that the necrotic tissue in the uterus served as the source of infection.
